# A Case of Malignant Airway Obstruction Mimicking Bronchial Asthma

**DOI:** 10.1002/rcr2.70169

**Published:** 2025-03-25

**Authors:** Yuka Matsuo, Yuki Takigawa, Ken Sato, Kenichiro Kudo, Hiromi Watanabe, Akiko Sato, Keiichi Fujiwara, Takuo Shibayama

**Affiliations:** ^1^ NHO Okayama Medical Center Department of Respiratory Medicine Okayama Japan

**Keywords:** AERO stent, airway stenosis, bronchial asthma

## Abstract

Asthma and central airway stenosis are often misdiagnosed due to overlapping clinical features, leading to delayed diagnosis and inappropriate management. A comprehensive diagnostic approach is crucial for accurate differentiation and improving patient outcomes.

## Graphical abstract

Central airway stenosis can be misdiagnosed as asthma. We present the case of a 63‐year‐old woman with breast cancer and a history of bronchial asthma who repeatedly presented to the emergency department with progressive dyspnea and who was initially misdiagnosed with asthma exacerbations. After eventual referral to our department, we report our comprehensive diagnostic approach and subsequent treatment that led to the accurate diagnosis of central airway stenosis and improvement of her condition and resolution of the stenosis. A comprehensive diagnostic approach is crucial for accurate differentiation and improving patient outcomes.

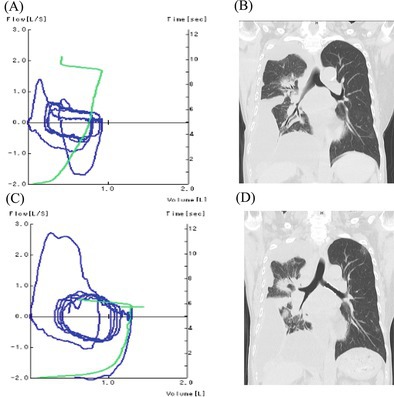

## 1 Clinical Image

**FIGURE 1:**
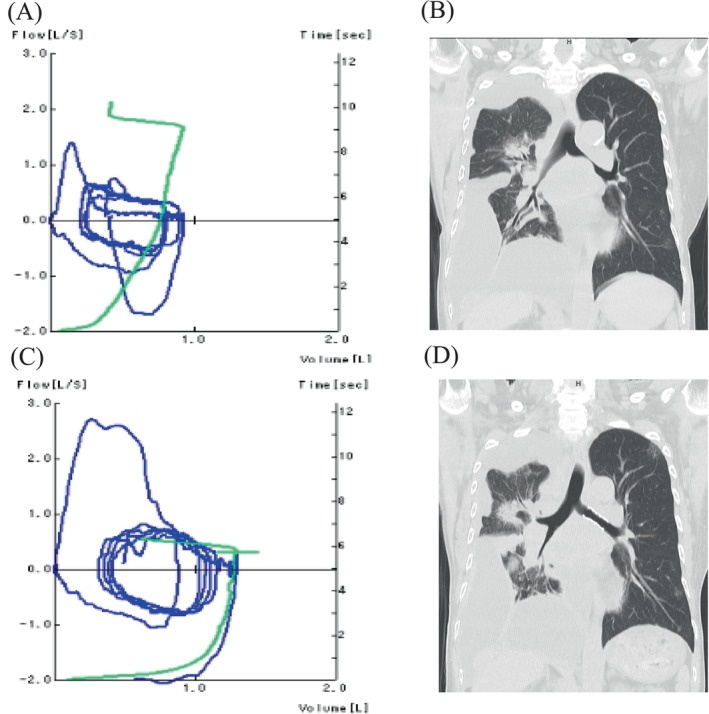
On admission, pulmonary function tests revealed a decreased FEV1% and reduced peak expiratory flow (A), indicating obstructive ventilatory dysfunction. Chest computed tomography (CT) performed the following day showed stenosis of the left main bronchus and right intermediate bronchus due to lymph node metastases. Additionally, drug‐induced pneumonia with right‐sided pleural effusion and ground‐glass opacities accompanied by infiltration in the right lung were observed (B). Following the intervention, pulmonary function tests revealed improvements in FEV1% and peak expiratory flow (C). Post‐intervention CT confirmed improvement in left main bronchial stenosis after stent placement (D).

**FIGURE 2:**
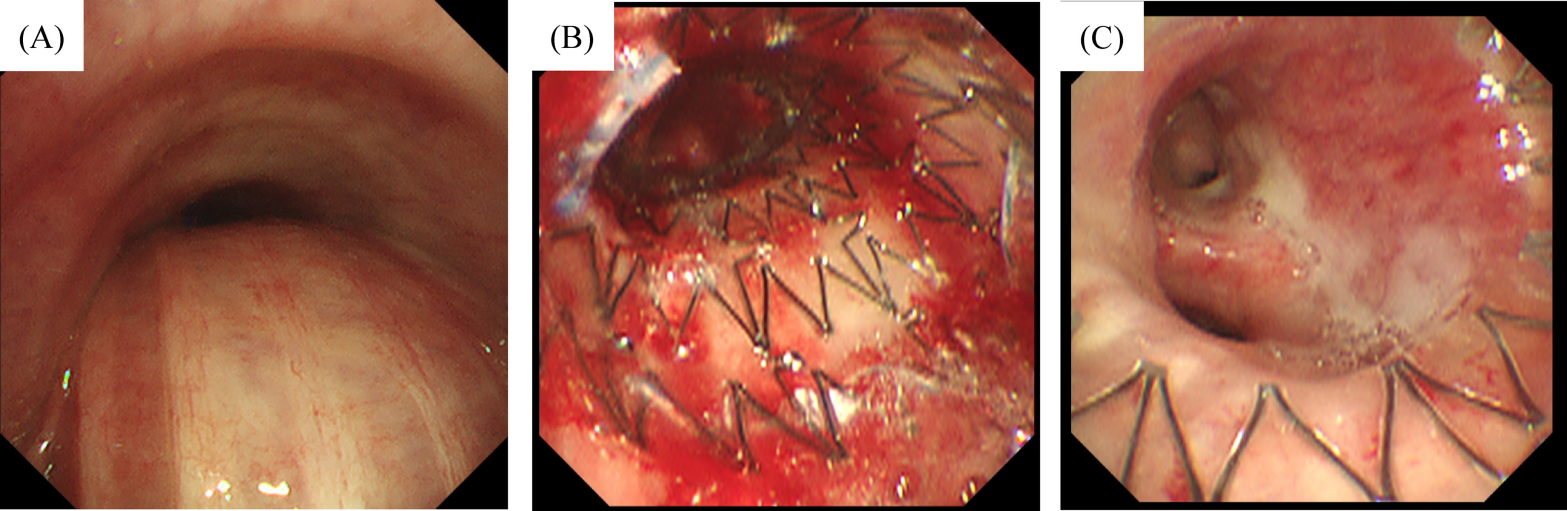
Flexible bronchoscopy revealed 90% stenosis of the left main bronchus, making visualisation of the distal airway impossible (A). Bronchial balloon dilation was performed, followed by the placement of an AERO stent (10 mm × 30 mm) (B, C).

Central airway stenosis can be misdiagnosed as asthma [1, 2]. A 63‐year‐old woman with breast cancer presented to the emergency department with progressive dyspnea for several times. She had been undergoing chemotherapy for recurrent breast cancer and started trastuzumab deruxtecan 2 months earlier. With a history of bronchial asthma, her symptoms were initially misdiagnosed as an asthma attack and temporarily improved with a short‐acting beta‐agonist. However, due to persistent symptoms, she was referred to respiratory medicine. Pulmonary function tests revealed an obstructive pattern (Figure 1A), with decreased forced expiratory volume in 1 s (FEV1%) and reduced peak expiratory flow, indicating obstructive ventilatory dysfunction. Central rhonchi on auscultation raised suspicion of central airway stenosis. Chest computed tomography (CT) revealed left main bronchial stenosis caused by lymph node metastasis, along with drug‐induced pneumonia, right‐sided pleural effusion, and ground‐glass opacities in the right lung (Figure 1B). Palliative pulmonary intervention with an AERO (Merit Medical Systems, South Jordan, UT, USA) stent was performed (Figure 2A), resulting in significant respiratory function improvement (Figure 2B,C). Follow‐up pulmonary function tests showed marked improvement (Figure 1C), and postoperative CT confirmed resolution of the stenosis (Figure 1D). This case highlights the importance of distinguishing malignant central airway obstruction from asthma to ensure proper management.

## Author Contributions

Yuka Matsuo and Yuki Takigawa prepared the manuscript, which was reviewed by all co‐authors.

## Ethics Statement

The authors confirm that appropriate written informed consent was obtained for the publication of this manuscript and accompanying images.

## Conflicts of Interest

The authors declare no conflicts of interest.

## Data Availability

The data that support the findings of this study are available from the corresponding author upon reasonable request.
